# DNA Methylation and Apoptosis Resistance in Cancer Cells

**DOI:** 10.3390/cells2030545

**Published:** 2013-07-18

**Authors:** Eric Hervouet, Mathilde Cheray, François Marie Vallette, Pierre-François Cartron

**Affiliations:** 1 Laboratoire de Biochimie, EA3922, UFR-ST, Université de Franche-Comté, 25035 Besançon Cedex, France; E-Mail: eric.hervouet@univ-fcomte.fr; 2 Centre de Recherche en Cancérologie Nantes-Angers, INSERM, U892, Equipe Apoptose et progression tumorale, Equipe labellisée Ligue Nationale Contre le Cancer, 44007 Nantes, France;; 3 Université de Nantes, Faculté de Médecine, Département de Recherche en Cancérologie, IFR26, F-4400, Nantes, France; E-Mail: mathilde.cheray@univ-nantes.fr; 4 LaBCT, Institut de Cancérologie de l'Ouest, Nantes, Saint Herblain Cedex, France; E-Mail: Francois.Vallette@univ-nantes.fr

**Keywords:** apoptosis, DNA methylation, cancer, epigenetic

## Abstract

Apoptosis is a cell death programme primordial to cellular homeostasis efficiency. This normal cell suicide program is the result of the activation of a cascade of events in response to death stimuli. Apoptosis occurs in normal cells to maintain a balance between cell proliferation and cell death. A deregulation of this balance due to modifications in the apoptosic pathway leads to different human diseases including cancers. Apoptosis resistance is one of the most important hallmarks of cancer and some new therapeutical strategies focus on inducing cell death in cancer cells. Nevertheless, cancer cells are resistant to treatment inducing cell death because of different mechanisms, such as DNA mutations in gene coding for pro-apoptotic proteins, increased expression of anti-apoptotic proteins and/or pro-survival signals, or pro-apoptic gene silencing mediated by DNA hypermethylation. In this context, aberrant DNA methylation patterns, hypermethylation and hypomethylation of gene coding for proteins implicated in apoptotic pathways are possible causes of cancer cell resistance. This review highlights the role of DNA methylation of apoptosis-related genes in cancer cell resistance.

## 1. Introduction

Apoptosis or programmed cell death is a key suicide program dominant in the maintenance of cellular homeostasis and is initiated by the activation of a cascade of events preexisting within each cell, in response to death stimuli. Apoptosis contributes to a balance between cell proliferation and cell death in normal cells and disturbances in this pathway are often observed in human diseases. Among the hallmarks characterizing tumor escape (tumor reccurence despite resection and treatments) [2], apoptosis resistance is one of the most striking. Furthermore, even if new therapeutical strategies have emerged over the last decades to force cancer cells to undergo cell death, apoptosis resistance to treatment is still a major problem in cancer research and requires extensive research to improve our knowledge of apoptosis signaling. Cancers cells have developed numerous strategies of resistance to cell death such as DNA mutations in genes coding for pro-apoptotic proteins, increase expression of anti-apoptotic proteins and/or pro-survival signals, or discovered more recently, pro-apoptic gene silencing mediated by DNA hypermethylation. DNA methylation occur in about 50% CpG in the genome and results from a transfer of a CH3 (methyl group) from S-adenosyl methionine to the fifth carbon of cytosine in CpG motifs. CpG-rich areas referred as CpG islands (CGI) are more frequently methylated than isolated CpG and is generally associated with gene repression. Two distinct mechanisms of DNA methylation have been reported. The first is maintaining DNA methylation, which occurs on neosynthesized strands of DNA following replication and is mainly catalyzed by DNA methyltransferase 1 (Dnmt1) [3]. The second process is *de novo* (dn) methylation, which occurs on both strands of unmethylated DNA and is predominantly catalyzed by Dnmt3a and Dnmt3b [4]. Epigenetics appears highly implicated in gene dysregulation in cancers. Many cancers are characterized by global DNA hypomethylation [5,6], previously associated with chromosomal instability, and paradoxically with both local hypo and hypermethylations [7,8,9]. It is now well known that local hypomethylation may lead to abnormal activation of genes including oncogenes while hypermethylation could silence tumor suppressor genes (TSG) [10,11]. Similar phenomenon occur in genes regulating the apoptosis pathway and these epigenetic modifications could influence the balance between pro- and anti-apoptotic protein expression conferring a phenotype of apoptosis resistance in cancer cells. Indeed, hypermethylation of a number of genes implicated in the apoptosic pathway has been reported in cancer cells (Table 1). Among them, some seem more specific to a tumor type while others appear commonly methylated in cancers as illustrated by the methylation of *Puma* in lymphoma [12] while *DAPK* or *RASSF1A* methylations have been found in most cancers analyzed (Table 1). Moreover, hypermethylation of these genes could occur sporadically in tumors or cover a large sequence of the promoter and are found from low to high frequencies in cancer patients (Table 1). Owing to pleiotropic effects of p53 silencing in cancers, methylation of this gene has not been considered in this review (For a review, cf [13]).

**Table 1:** Apoptosis related genes are frequently methylated in tumors. Percentage of methylated tumors is indicated in brackets for significant histochemical studies.

Gene methylated	Cancer	Ref
APAF-1	Melanoma, Leukemia, Testicular (100 *vs.* 60), Bladder (11), RCC (100)	[14], [15], [16], [17], [18]
Bad	Myeloma	[19]
Bak	Myeloma	[19]
Bax	GBM, Myeloma	[20], [19]
Bcl2L10	Gastric (38), Leukemia (12-45)	[21], [22]
Bim	CML	[23]
Bik	Glioma (30), HCC, RCC, Prostate, Myeloma	[24], [25], [26], [27]
BNIP3	Pancreatic, Gastric (39), Breast, Colorectal, Leukemia, Myeloma, HCC	[28], [29], [30], [31]
Casp-8	Medulloblastoma (62–81), Pituitary tract (54), Rhabdosarcoma (83 vs 0), Phaeochromocytoma (31), Neuroblastoma (35-52 vs 0), Retinoblastoma (59 *vs.* 0), HCC (34), GBM (30), Bladder (19), Lung (0–45 *vs.* 0), Rectal, Breast, Prostate, Gastric	[32], [33], [34], [35], [36], [37], [38], [39], [40], [41]
DAPK	Mesothelioma (20), Testicular (20–50 *vs.* 6), Nasopharyngial (76 *vs.* 0), Pituitary (43), Colorectal (81), ACC (27), Lung (25–44), Biliary tract (21), Lymphoma (71–85), GBM (14), Gastric (22–70), Leukemia (36), Breast (13–88), CXCA (56–79), Cholangiocarcinoma (31), Bladder (74–77), RCC (33–55), Head and Neck (11–33), Myeloma (40), Oesophageal (50–60 *vs.* 20), Ovarian	[42], [43], [44], [45], [46], [47], [48], [49], [16], [50], [36], [51], [52], [53]
DcR1-2	Glioma (60), Neuroblatoma (11–25), Prostate (50), Breast, Prostate (37–45), Ovarian (31–43), Breast (70), Lung (31), Mesothelioma (63), Bladder (42), CXCA (100), Lymphoma (41), Leukemia (26), Myeloma (56), Phaeochromocytoma (23–26)	[54], [32], [55], [56], [57], [58], [37]
DR4 or 5	Breast, Melanoma, Ovarian (10–28), Phaeochromocytoma (41), Neuroblastoma	[54], [59], [60], [37], [61]
Fas	Lymphomas, CXCA, Colon, Prostatic (12), Lung	[62], [63], [64], [65]
Hrk	Colorectal (36), Gastric (32), GBM (27–43), PCNSL (31), Prostate (38)	[66], [67], [68], [69]
Puma	Lymphoma	[12]
RASSF1a	ACC (42–45), Biliary tract (27), CRCC (45), Nasopharyngeal (71–84 *vs.* 0), Ovarian (26), Gastric, Bladder (48–60 *vs.* 42), Thyroid, Neuoblastoma (83–93), Osteosarcoma (14 *vs.* 1), Lung (43 *vs.* 5), Breast (63–74), , HCC Hepatoblastoma (39–44), CXCA (26 *vs.* 0), Mesothelioma, Glioma (12–82), Endometrial, Liver (38), Prostate, Parathiroid (98), Lung (26), Testis (78 *vs.* 0), Melanoma (69), Colorectal (31), Retinoblastoma (98)	[70], [71], [72], [73], [74], [75], [76], [32], [77], [78], [79], [80], [81], [82], [56], [83]
SARP2	Pancreatic (90–90 *vs.* 0–5), Colorectal, Oesophageal	[84]
TMS1	Cholangiocarcinoma (36), Prostatic (47–65), Colorectal (25–41 *vs.* 8), Ovarian (19 *vs.* 0), Thyroid (33), Breast (24–46), Gastric (32), GBM (21–57), Lung (41–70), Melanoma, Colorectal, Neuoblastoma, HCC, Pancreatic	[85], [86], [87], [88], [89], [55], [90], [91], [92], [93], [94]
TNFR10c	Pancreatic (54–97), Choroid plexus (50), Neuroblastoma (21), Breast (48), Lung (37), Mesothelioma (43), Ependymomas (50)	[95]
XAF-1	Colorectal (40), Gastric, Bladder, RCC, Prostatic (35 *vs.* 0), Lung, GBM (22), Oesophageal	[96], [97], [98], [99], [34], [100]

## 2. Apoptotic Pathways

**Figure 1:**
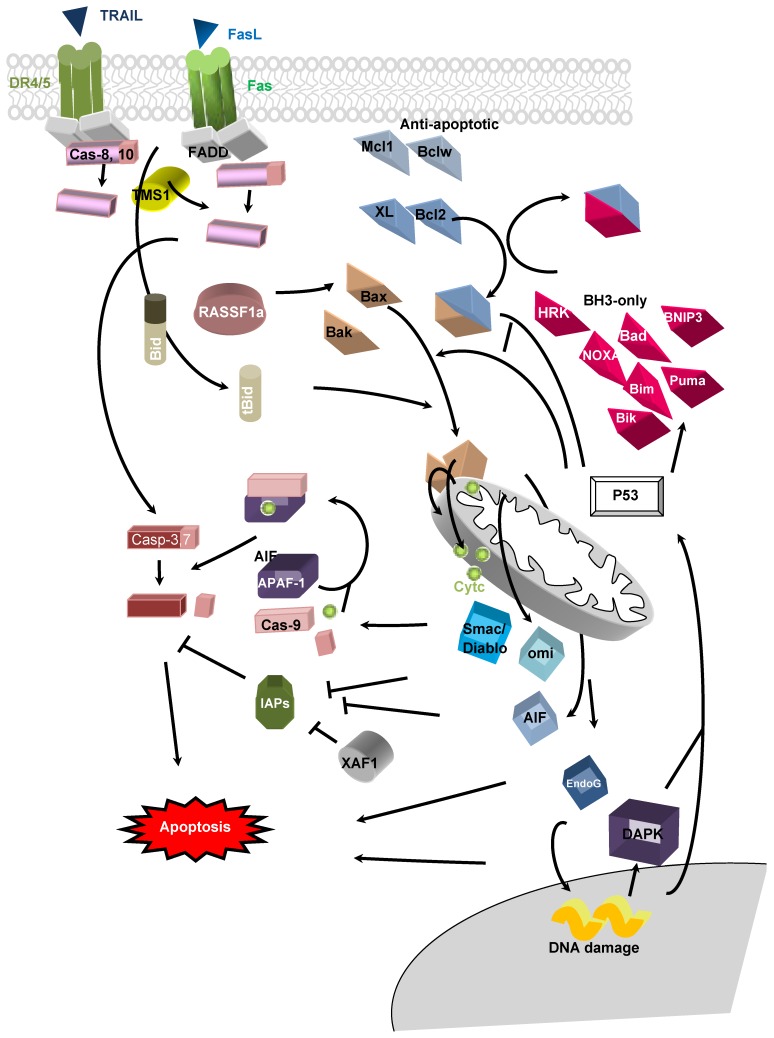
Mechanism of apoptosis. Activation of initiators caspase 8 and 10 are mediated in response to extrinsic stimuli and apoptosis signaling is controlled by anti- and pro-apoptotic protein interactions.

Both the intrinsic and extrinsic pathway of apoptosis have been reported in mammals (Figure 1). Briefly, the intrinsic pathway involves a diverse array of non-receptor-mediated stimuli that produce intracellular signals that act directly on targets within the cell and are mitochondrial-initiated events which can be activated by the TSG p53 in response to cell injury such as DNA damage following radiation exposure. The Bcl2 protein family is composed of pro-apoptotic and anti-apoptotic members and are classified in three subfamilies with regards to their Bcl2 homology domains (BH). Bax and Bak present 3 BH domains (BH1-3) and are associated with pro-apoptotic properties. Conversely, anti-apoptotic proteins Bcl2, BclXL, Mcl-1, A1 and Bcl-W present 4 BH domains (BH1-4), and are capable of antagonizing Bax and Bak. Bim, Bik, Bad, Hrk, Noxa and Puma, which belong to the third subfamily (referred to BH3-only) and are devoided of BH1, 2 and 4. BH3-only proteins are also able to bind pro-apoptotic proteins (like Bax and Bak) and activate or block their activity. Following the initiation of apoptosis, Bax and Bak undergo a conformational change, which consequently induces a permeabilization of the outer mitochondrial membrane releasing several mitochondrial proteins from intermembrane space (cytochrome c, Smac/diablo, HtrA2/omi). Interaction of APAF-1 with cytochrome c and proCasp 9 leads to the formation of the Apoptosome complex and the activation of Casp 9. Finally, cleavage of effector proCasp3 and pro-Casp7 into active Casp3 and 7 and nuclear fragmentation causes the formation of apoptotic bodies. The extrinsic pathway is activated in response to cytokines. Tumor necrosis factor-related apoptosis-inducing ligand (TRAIL or Apo2 ligand) is able to selectively induce apoptosis in tumor cells with no significant untoward effect on normal cells. Apoptosis induced by TRAIL is initiated by its binding to death receptors (TRAIL-R1 or DR4 and TRAIL-R2 or DR5) followed by formation of the death-inducing signalling complex (DISC) upon recruitment of specific cytoplasmic proteins, Fas-associated death domain (FADD) and caspase-8 or -10. Binding of FasL to the extracellular area of Fas (APO1/CD95) induces a conformational change in the receptor and a recruitment of FADD (Fas-interacting DD) and proCasp 8 and proCas 10 effectors in the cytoplasmic region of the receptor *via* its death domain. Formation of this complex induces an increased concentration of initiator caspases: proCasp 8 and 10 on Fas receptor and their automatic cleavage to active Casp 8 and 10. Once activated, these caspases can directly activate Casp 3 or connect to the intrinsic pathway by the cleavage of BID into tBID catalysed by Casp 8, which consequently leads to the activation of Bax and mitochondrial pathway and further Casp 3 activation.

## 3. DNA Hypo/Hypermethylations in Apoptosis-Related Genes in Cancer

### 3.1. Extrinsic Pathway

#### 3.1.1. Death Receptor

Aberrant methylation in the promoters of gene coding for proteins implicated in the extrinsic pathway could be responsible of early blocking events in apoptosis. A decrease in Fas expression has been observed in p53 mutated cancer cells. Two distinct regions controlling *Fas* expression are sensitive to methylation. The Fas promoter contain a 650 pb CGI (28 CpG sites) in the 5’flanking region and a second CGI in intron 1. Such aberrant methylation has been reported in *Fas* promoter in T-cell lymphoma or in colon carcinoma cell lines and also correlated to a decrease in Fas expression, thus conferring apoptosis resistance independently of the p53 status. Conflicting results have been reported on the correlation between methylation and *Fas* gene silencing. Butler *et al.* [101] proposed that in colorectal carcinomas *Fas* silencing might not be due to promoter methylation as they did not find any methylation in the 9 CpG located in the *Fas* promoter. However, it should be noted that this study did not include a large area of the promoter, such as the CpG site at -548 position, the only CpG methylated in RKO cells, the demethylation of which by 5-azadeocytidine restored Fas expression and apoptosis. Specific methylation of p53 binding site in intron 1 may also explain the efficient *Fas* silencing in some colon cancers [64]. Aberrant methylation in the promoters of *DcR1, DcR2*, *DR4* and *DR5* have been reported in neuroblastoma tissue or cell lines and inversely correlated to expression of these genes. TNFRSF10c belongs to the tumor necrosis factor receptor family and has also been frequently deleted in many cancers but silencing of the *TNFRSF10C* gene in pancreatic cancers occur predominantly by hypermethylation in CpG islands associated with apoptosis resistance [95].

#### 3.1.2. Caspase 8 (Casp 8)

Whereas, according to literature, methylation of *Fas* or *DcR/DR* genes are rare events, many reports have shown the frequent hypermethylation of the promoter of *Casp 8* in many different cancers (Table 1). Methylation in the *Casp 8* promoter generally correlated with a low Casp 8 expression, and low apoptosis sensitivity to trail and was reversed in different cancer cell lines as lung cancer cells, using a 5-azadeoxycytidine treatment, an inhibitor of DNA methylation. While mechanisms governing genes hypermethylation in cancers—in particular in apoptosis pathways are until now poorly understood—some initial results have been proposed to explain the hypermethylation of the *Casp 8* promoter. Kurita *et al.* [102] reported that both invalidation of Dnmt1 and Dnmt3b was required to reverse *Casp8* promoter methylation and induce gene reexpression in human hepatoma cells. However, we recently reported that *Casp8* methylation correlated with both Dnmt1 and Dnmt3a, but not with Dnmt3b [34]. These reports strongly support a role for cooperation between Dnmt1 and *dn* Dnmts in *Casp8* methylation. The requirement of Dnmt3a or Dnmt3b might be governed by tissue or CpG specificity. Dnmt1/Dnmt3b cooperation has already been described for methylation of survival genes (*p16*, *BAGE1*, *CXCL12*). Molecular mechanisms at the origin of *Casp8* methylation have been partially analyzed. The methylation in the SP1 box located at -97 position completely abolished SP1 complex recruitment and Casp 8 expression [103]. Similarly, in neuroblastoma, 63% of tumors presenting c-MYCN oncoprotein amplification were also associated with methylation of *Casp 8* whereas only 6% of tumors without c-MYCN amplification were methylated [104]. With regards to the new roles of TFs (Transcription Factors)/Dnmts interaction in gene silencing, these observations might suggest a role for SP1 and/or c-MYCN in *Casp8* methylation. Moreover, few regulators of the extrinsic pathway are known. Methylation-dependent silencing of these proteins could also play a role in apoptosis resistance in cancer cells.

#### 3.1.3. TMS1/ASC (target for methylation-induced silencing-1/apoptosis-associated speck-like protein containing CARD-ASC).

A CGI gene called *TMS1/ASC* encodes for a pro-apoptotic tumor suppressor protein composed of PYD (pyrine domain) and CARD motifs. Activation of TMS1 is able to induce apoptosis in a Casp 8-dependent manner (Figure 1). A high frequency of methylation was found in *TMS1* promoter in many tumors and the density of methylation inversely correlated both, in tissue and cell lines, to TMS1 expression as illustrated in glioma cells [105]. Invalidation of *TMS1* in cultured cells induced a partial protection against TRAIL-induced p53-mediated apoptosis, however, contribution of *TMS1* methylation to cancer progression is not clear as no correlation could be found between methylation and pathological changes in several cancers including cholangiocarcinoma [88,106]. Since *TMS1* methylation in normal tissue adjacent to prostate tumors was significantly more frequent in patients with biochemical recurrence, the *TMS1* methylation event was suggested to contribute to aggressiveness in some cancers. The latter observation was validated in neuroblastoma patients showing links between *TMS1* methylation and shorter survival curves [85,107]. *TMS1* mediated-methylation was significantly higher in clear cell ovarian tumors than in other ovarian cancer types, suggesting different roles of TMS1 inactivation in tumors from different tissues but also from the same organ [90]. Interestingly, Das *et al.* [108] reported a significant difference in methylation frequencies in *TMS1* promoter in prostate cancers based on racial criteria (black or white) associated with a mortality ratio two-fold higher in the black male population. Environment, diet and ethnic habits are supposed to influence methylation status of genes and might contribute to epigenetic-mediated tumorigenesis. Silencing machinery controlling *TMS1* methylation has been poorly studied. *TMS1* silencing could result from dense methylation in CGI combined with hypoacetylation in H3 and H4 and the modification of the chromatin structure in CGI. In glioma patients, *TMS1* methylation was correlated with Dnmt3b content and overexpression of Dnmt3b caused an increase in *TMS1* methylation, while Dnmt3a overexpression had no effect. In glioma cells, corecruitment of Dnmt1, Dnmt3b and HDAC1 on the *TMS1* promoter suggest a silencing complex requiring a cooperation between DNA methylation and hypoacetylation [34]. While no association was found between Dnmt1 and TMS1 expression, Dnmt1 is a member of the silencing complex; moreover, overexpression of Dnmt1 in fibroblast was also silenced in a DNA methylation manner the *TMS1/ASC* gene [109]. Indeed, recruitment of MBD3 on the *TMS1* promoter in prostate cancers also suggested a role for the the NuRD complex in *TMS1* silencing.

### 3.2. Intrinsic Pathway

#### 3.2.1. Bax

**Figure 2:**
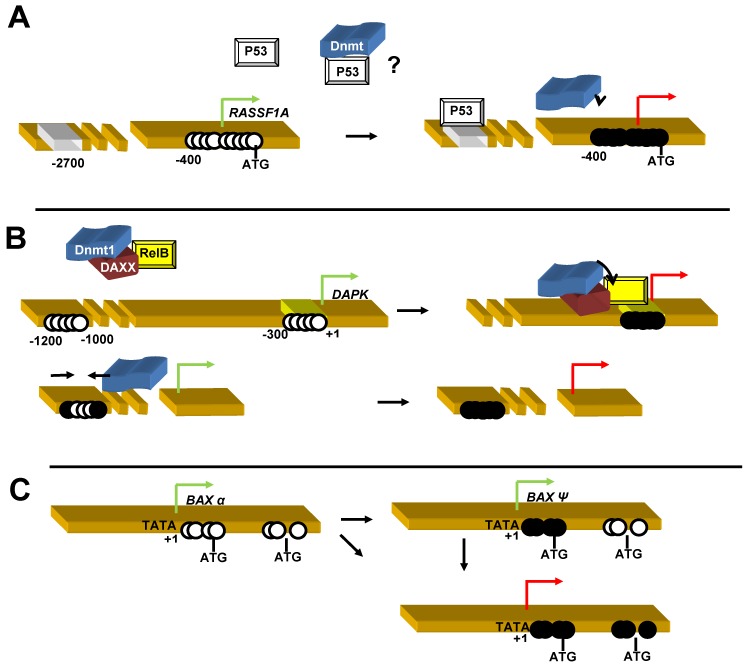
Mechanisms of DNA hypermethylation and apoptosis-related genes inactivation (**a**) Inactivation of *RASSF1A* by Dnmt/P53 cooperation. (**b**) Inactivation of *DAPK* by the ternary complex Dnmt/RelB/DAXX. **(c)** Specific methylation of *BAX* leading to BAX ψ or BAX silencing.

Methylation in the Bax promoter induced a strong decrease or complete silencing of Bax expression in glioma cells or glioma patients. Moreover, silencing of Bax by RNAi conferred a resistance to Fas ligand-mediated apoptosis in cell culture suggesting a role of methylation-mediated Bax silencing in apoptosis resistance in GBM. The low number of GBM patients presenting Bax silencing does not permit a serious statistical survival analysis [20,34]. However, it should be noted that in a cohort of 27 GBM patients, the patient presenting a Bax inactivation via methylation had the lowest survival curve. Interestingly, our group reported another distinct methylation profile of *Bax,* which was called *BaxΨ* and this methylation profile correlated with an extended survival curve in glioma patients [20]. BaxΨ isoform was induced by methylation within intron 1 of the gene and induced the expression of a truncated form of Bax (Bax^p18^), which was more apoptogenic than Bax^p21^ (Figure 2C). Both intron 1 and exon 1 methylations were observed in non expressing Bax cells, suggesting that BaxΨ profile might be an intermediary step in *Bax* silencing.

#### 3.2.2. Bcl2

Surprisingly, a frequent hypermethylation of two CpG in the Bcl2 promoter was observed in prostate cancer and was associated with a decrease in Bcl2 expression [110]. The implication of *Bcl2* methylation in prostatic cancer is not understood and has to be balanced by the additional decrease expression of pro-apoptotic protein such as Bak and Bik in these cancers. In glioma patients, *Bcl2* methylation correlated both with global DNA methylation as well as another methylation of the anti-apoptotic gene *BclXL,* suggesting common methylation mechanisms and this was associated with an increase in intra tumor apoptosis [34]. 

#### 3.2.3. BH3-only

BH3-only genes encode for pro-apoptotic proteins capable of sequestering anti-apoptotic proteins such as Bcl2 or BclXL (Figure 1). Several BH3-only genes are methylated in cancers. A decrease in HRK (Harakiri) expression was found in different cancers including prostate or glioma cancers and was associated with apoptosis resistance. HRK gene inactivation frequently resulted from a combination of an initial loss of one allele and a further methylation of the second allele [111]. Surprisingly, *HRK* methylation was significantly higher in gastric tumor or glioma patients presenting WT p53 suggesting a role of this protein in the specific methylation of this gene. Literature suggests that *HRK* methylation is not always correlated to a decrease in *HRK* expression. Indeed, some large regions, which are very frequently methylated, seem to be those which are the least associated with silencing, while a dense methylation of few CpG closed to transcriptional initiation site abrogated *HRK* expression [66]. Finally, deacetylation of H3 and H4 also regulate *HRK* silencing. Specific methylation of *Puma* gene have been reported and associated to gene silencing in lymphoma while *BIM* was methylated in CML [11,32,33]. *BIK* inactivation in a mutational manner is observed in lymphomas, but epigenetic modification seems to be also implicated in *BIK* silencing in other cancers as Multiple Myeloma [27]. *BIK* is methylated in glioma and in several cell lines but studies on a direct link between methylation and low expression is still under discussion. Nevertheless, inhibitors of DNA methylation in RCC cell lines induces a strong increase in BIK expression and abrogated apoptosis resistance. A synergic increase in both mRNA and BIK protein in HCC cells was observed following treatment with both inhibitors of Dnmt and HDAC suggesting a cooperation between deacetylation and DNA methylation occurs in BIK silencing [24,112].

#### 3.2.4. BCL2L10

BCL2L10 belongs to the Bcl2 family but some conflicting results described this protein as pro or antiapoptotic. Yet, Xu *et al.* [21] reported that three sites where hypermethylated and three additional sites were partially methylated in *BCL2L10* promoter in gastric tumor tissue and correlated with a decrease in the corresponding protein. Moreover, silencing of the gene was correlated to an increasing tumor size in these patients.

#### 3.2.5. APAF-1 (Apoptotic Protease-Activating Factor 1)

APAF-1 silencing is frequently observed in melanomas and is partly due to LOH. As 5-aza treatment dramatically increased APAF-1 expression in methylated melanoma or leukemia cells but not in bladder cells, the link between APAF-1 methylation and gene silencing is not clear and might be tissue-dependent [18]. *APAF-1* methylation correlated with tumor grade in bladder cancers, RCC or neuroblastoma, but while a significant increase of *APAF-1* methylation was also observed in testicular germ cells tumors, the relative abundant basal *APAF-1* methylation in non tumoral tissue leads to a difficult interpretation of its role in tumorigenesis [16,85,113]. Since no correlation between *APAF-1* mRNA and APAF-1 protein content could be found, post-translational modification of APAF-1 are suspected to be dramatically involved in apoptosis sensitivity [114]. However, inhbition of *APAF-1* methylation by 5-aza deoxycytidine, restores the Apoptosome formation and the activation of Casp 9. On one hand, specific methylation of CpG were found in a region from +87 to +128 in leukemia and not in normal cells, and on the other hand, no modification in the methylation rate in CGI (−680 +420) of the gene could be found between melanomas and normal cells, leading to the idea that methylation of an enhancer, still unknown until now, could also be implicated in the phenomenon [17]. Finally, an increase in Dnmt1 content is suspected to favor *APAF-1* methylation whose complete silencing probably requires the recruitment of the additional corepressors HDAC1 and MBD2.

#### 3.2.6. Inhibitor Apoptis Proteins (IAPs)

Inhibitor Apoptosis Protein (IAP) is composed of eight members presenting from one to three BIR domain from the baculovirus. IAPs antagonized effectors caspases : Casp 3, Casp 7 and Casp 9, and inhibit apoptosis. A loss of methylation in these promoters might be related to apoptosis resistance. Five *IAP* promoters (ciAP1, ciAP2, Survivin, NAIP and XIAP) were hypomethylated in oral tumors and a strong correlation of expression between each other suggests some common regulatory mechanisms. However, any correlation between IAP expression and malignant transformation in oral tumors was found, suggesting that a gain in expression of *IAP* genes might only partially contribute to tumorigenesis and not be at its origin [115]. Survivin is normally expressed during embryogenesis and shut down in adult differentiated tissues. Strikingly, *Survivin* hypomethylation and high overexpression of the protein has been found in different cancers including ovarian, HCC(Hepato Cellular Carcinoma) and glioma. A correlation between Dnmt1 level and *Survivin* methylation status suggest that Dnmt1 is required for *Survivin* regulation. Esteve *et al.* [116] proposed that Survivin silencing was mediated by a heterotrimer SP1/Dnmt1/p53 complex initiated by TFs/Dnmts interactions and specific recruitment of Dnmt1 on TF-Boxes.

#### 3.2.7. BNIP3

The TSG, BNIP3 (Bcl2/adenovirus E1B 19KDa interacting protein 3) is normally activated by HIF (hypoxia inducible factor) in response to hypoxia and can promote apoptosis. As chronic hypoxia is a hallmark of environment of many solids tumors, cancers have frequently developed strategies of resistance, and among them, the monoallelic deletion of BNIP3. Indeed, CpG islands were found from the 5' end to the intron 2 (−1162/+538) of *BNIP3* gene and methylation in these sites correlated with low expression of BNIP3 in pancreatic tumors where most of the CpG analyzed were specifically methylated in tumoral tissue [117]. Silencing of the gene could be explained by the presence of 4 SP1 motifs in the methylated region and methylation in the vicinity of HRE binding site in pancreatic tumors. Relationships between inactivation of *BNIP3* gene and survival is confusing. However, forced expression of BNIP3 in pancreatic tumoral cell lines leads to apoptosis. Survival time of patients with *BNIP3* methylation was shorter than in absence of methylation in gastric tumors, whereas no such association could be found in breast tumors [28]. In addition, patients with methylation exhibited resistance to chemotherapy compared to patients with no methylation, suggesting that methylation of BNIP3 is a predictive factor in the prognosis and response to treatment in colorectal cancer patients [118,119,120].

#### 3.2.8. XAF-1

The pro-apoptotic protein XAF-1 (XIAP-associated Factor 1) is the main inhibitor of XIAP by inducing its sequestration (Figure 1). No canonical CGI could be found in promoter but two shorts regions (I and II) quite rich in CpG were found within -1867/-343 area but methylation in both I and II appeared not critical for XAF-1 expression in gastric cancers [99]. These authors demonstrated that XAF-1 expression was rather tightly controlled by the methylation status of seven independent CpG localized in the proximity of the transcriptional initial start site (-234/-23). Common and additional methylated sites in -164 to 109 were found in colon carcinoma cell lines suggesting tissue-specific mechanisms of inhibition particularly the two CpG located to the transcriptional start site [121]. Indeed, similar results from Lee *et al.* reported that *XAF-1* hypermethylation of total or partial 14 CpG loci from -20 to -695 of the promoter, was associated to a decrease in XAF-1 expression and is correlated with disease progression, in particular malignancy in bladder cancers [122].

#### 3.2.9. Death Associate Protein Kinase (DAPK)

Death associated protein kinase (DAPK) gene encodes for a Ca+/calmodulin-regulated Ser/Thr kinase including a death domain and is involved in both intrinsic and extrinsic apoptosis. DAPK promoter is the most frequently apoptosis-related gene aberrantly methylated in cancers at frequencies often from 70–90%. Methylation of *DAPK* promoter is clearly associated to disease in many cancers. As some examples, patients with *DAPK* methylation in gastric cancers present a response rate significantly lower than patients without methylation and *DAPK* methylation also correlated with grade in testicular tumors, recurrence in bladder tumors, or invasion and metastasis in lung cancers [93,98,123]. Conversely, any influence of *DAPK* methylation could be found on prognosis in malignant mesothelioma as neither CXCA patients and overexpression of DAPK increased the radiosensitivity of SiHa cells [42,46]. As methylation correlated to low DAPK expression, Mittag *et al.* [51] suggested that methylation of *DAPK* conferring apoptosis resistance was an early event in tumorigenesis and several molecular explanations have been proposed. *DAPK* gene present a CGI extending 2500 pb from the transcriptional start site. A high heterogeneity of methylation of CpG was found in lung cancers suggesting that some CpG are more particularly associated with gene silencing than others. Methylation in the intron 1 partially correlated to DAPK expression while those included in 5’flanking region are more related to DAPK silencing [53]. In cancer cells, edge of CGI is more sensible to methylation than the center but is not associated to gene expression regulation [124]. It has been hypothesized that initial methylation of edge of CGI, particularly in a repetitive element, such as Alu sequences, could bring a functional anchorage of methylation machinery for further methylation toward the center of the CGI which would affect gene expression. Indeed, a frequent methylation in the exon 2 of cancer cells was not correlated to low expression. Pulling *et al.* [125] also recently revealed two methylation hot spots containing each 8-10 CpG whose methylation abolished DAPK expression. Methylation occurring in the vicinity of CP2 or FOXA2 binding sites negatively controlled the expression of the two promoters of DAPK. Concomitantly to DNA methylation, deacetylation of histones H3 and H4 was observed suggesting a complex including Dnmt and HDAC in *DAPK* silencing [124]. A role of DAXX on DNA hypermethylation has recently emerged. DAXX is a dual protein: i) implicated in apoptosis and ii) is a transcriptional regulator able to bind to HDAC and Dnmt to repress gene transcription. DAXX can not directly bind to DNA and Puto *et al.* [126] demonstrate that its repression activity was mediated via RelB interaction (Figure 2B). Formation of the ternary DAXX/Dnmt1/RelB complex transformed the transcriptional activity of RelB into a repressor complex by inducing a targeted methylation-mediated silencing on RelB-binding sequences in several genes including *DAPK*. Participation of chromatin remodeling enzymes was confirmed by the co-recuitment of HDAC2 on DAXX.

#### 3.2.10. Ras Association Doman Family 1A (RASSF1A)

Ras association domain family 1A (K-Ras/ RASSF1A) complex can activate Bax *via* an additional intermediately interaction with MOAP-1. In spite of absence of TATA box (also called Goldberg-Hogness box), methylation in CGI adjacent to the transcription initiation site induced gene silencing and *RASSF1A* methylation was considered has a good prognosis marker in breast, CRCC and some lungs cancers [127]. Some results in other cancers are less convincing, such as the absence of a correlation between methylation and clinical parameters or the observation that while *RASS1FA* is methylated at 48–60% in bladder cancers, a strong methylation (42%) was also found in normal corresponding tissue [128,129]. Interestingly, recent reports brought uncommon molecular mechanisms in *RASSF1A* methylation. Tian *et al.* [77] demonstrated that p53 was recruited on the p53 binding site located in distal promoter specifically in testis tumors but not in normal tissue and correlaled to DNA methylation in the proximal promoter (Figure 2A). A first recruitment of the HDAC1/SETDB1 (a histone methyltransferase) complex was supposed to initiate epigenetic marks on *RASSF1A* promoter. Secondly, *RASSF1A* methylation was achieved by the following recruitment of Dnmt3a favored by direct binding with both HDAC1 and SETBD1 [130]. Moreover, the ΔDnmt3b4 isoform of Dnmt3b lacking exon 6, appeared essential for *RASSF1A* silencing in a methylation manner in lung cancer, demonstrating for the first time the role of Dnmt isoforms in dn methylation in cancer cells [131].

#### 3.2.11. Secreted Apoptos-Related Protein 2 (SARP2)

Secreted Apoptosis-Related Protein (SARP) families are considered to counteract the oncogenic Wnt signaling pathway, and inactivation of this gene may aid cancer development and progression. SARP proteins, also named sFRP (for Secreted Frizzled-Related Protein), exert differential effects on osteoblastic differentiation of mouse mesenchymal cells and cellular apoptosis of mouse osteoblasts *in vitro* [132]. SARP2 methylation has been reported in several cancers such as pancreatic tumors with very high frequencies. SARP2 methylation may occur in the steps of pancreatic tumorigenesis as SARP2 methylation was observed in both begnin and malignant tumors and no statistical link could be found between SARP2 methylation and disease progression [84]. 

#### 3.2.12. Interferon Regulatory Factor 8 (IRF8)

Interferon regulatory factor 8 (IRF8) is an apoptosis-regulating gene that has been demonstrated to directly regulate Bax transcription *in vivo* [133]. IRF8 is hypermethylated in different types of cancer and is an essential regulator in Fas-mediated apoptosis pathway. Moreover, IRF8 is a metastasis suppressor in solid tumors and metastatic tumor cells use DNA hypermethylation to repress IRF8 expression to evade apoptotic cell death and to acquire a metastatic phenotype in human colon carcinoma [134]. IRF8 was also identified as a functional tumor suppressor, which is frequently silenced by epigenetic mechanism in multiple carcinomas [135]. Finally, silencing of IRF8 expression, by DNA methylation or other epigenetic mechanisms, may be associated with the malignant phenotype of Multiple Myeloma [136].

## 4. Significance of Methylation in Cancers

As seen above, mechanisms governing *de novo* methylation in apoptosis related genes are poorly understood and often completely unknown. A significant decrease in Dnmt3a content has been recently reported during TRAIL-induced apoptosis in rat hepatic cells [137]. It was generally believed that promoters with low CpG content were easily methylated but that they did not severely affect gene transcription. Similar hypotheses were proposed for a low percentage of methylation in high CpG regions. Nevertheless, in regards to increasing knowledge of DNA methylation mechanisms, reality may be much more complicated. Methylation of one site in 300pb in several promoters could be efficient to repress transcription. In some cases, specificity of Dnmt has been proposed, as the requirement of Dnmt3b or Dnmt1 for promoter methylation of *TMS1* or *Survivin,* respectively. Some recent works converging from several laboratories have demonstrated the existence of targeted methylation via Dnmts/TF interactions. The TF PU.1 physically interacts with Dnmt3a and mediated gene silencing via PU.1-binding sites in the promoters [55]. Similar observations with Dnmts/TF interactions have been described for p53 and cMyc [138,139]. 5-aza-deoxycitidine activates p53 signaling pathway and apoptosis cells [140]. As p53 is (i) a key regulator of apoptosis and (ii) control the transcription of many apoptosis related-genes, silencing via methylation of p53 binding sites by the Dnmt3a/p53 complex may confer a signature participating in apoptosis resistance. We demonstrated that TF-mediated targeted methylation could be a frequent phenomenon as Dnmt1, 3a and 3b potentially interacts with a large panel of TFs *in vitro* [141,142]. Methylation of SP1-binding sites has been reported in silencing of beclin. We recently validated Dnmt1/SP1 or Dnmt3/SP1 interactions. Modulation of theses complexes in tumoral cells could contribute to *de novo* methylation in apoptosis related genes promoters. Moreover, different Dnmt1/TFs complexes are observed in the different phases of a cell cycle, which means that targeted DNA methylation may be dependent on the accessibility of each TF [143]. EZH2 has been implicated in DAPK silencing. An increase of EZH2 expression in prostate and glioma cancers compared to normal tissue and a strong correlation between EZH2 level and bladder cancer stages may suggest that Polycomb group could partially control gene silencing observed in these tumors [15,70,144]. An illustration of this mechanism was the EZH2-mediated silencing of hDAB2IP, a GTPase-activating protein modulating apoptosis in prostate tumors. As APAF-1 expression is regulated by E2F1, SP1 and p53, further studies on possible mechanisms of targeted methylation and stabilization and kinetics of Dnmt/E2F1, Dnmt/SP1 or/and Dnmt/SP1 complexes may lead to understand the APAF-1 silencing in tumors.

We have seen above that numerous genes implicated in apoptosis could be aberrantly methylated in cancers and this was often associated to apoptosis resistance. Netherveless, hypermethylation of pro-apoptotic genes may not always associated with survival curve differences or grade. In CxCa (Cervical cancer), correlation of methylation have been found between *Fas* and *DAPK* in one hand and *TRAILR1* and *Fas* in another hand [63]. Authors suggested that high apoptosis resistance in tumor cells could resulted from the silencing of several apoptosis related genes issued from common or different mechanisms. Similarly, combined methylation of *TMS1* and *DAPK* in gastric cancers was associated to patients with a shorter survival curve in comparison to patients with only one methylation or no methylation [47]. Our recent work agree with these observations [34]: among eight genes related to the apoptotic program, only methylation status of *Bax* correlated to survival curve. As all these genes are included in a global cascade, we proposed that in glioma patients, each methylation could contribute to a small part in apoptosis resistance. A value of +1 was attributed to each tumor harboring a methylation in a gene coding a pro-apoptotic protein and −1 was attributed to each tumor harboring an unmethylation of an anti-apoptotic gene. Glioma patients scores revealed a strong correlation between score and apoptosis level and moreover, patients with the lowest score presented a significantly shorter survival curve. Similar clusters of gene methylation demonstrated a shorter survival in mesothelioma patients with combined methylation of *DAPK*, *RASSAF1A* and *RARβ* while no association was found for each methylation independently. Moreover, clustering of patients with three hypermethylated genes compared to unmethylated genes or one hypermethylated gene severly decrease the survival in neublastoma patients [85].

Recently, microRNAs (miRNAs) have been implicated in regulation of gene expression and of apoptosis-related gene expression. MiRNAs are small noncoding RNAs that function as endogenous post-transcriptional silencers of target genes. MiRNAs are expressed in a tissue specific manner and play important roles in cell proliferation, apoptosis and differentiation. Some miRNAs promoters are controlled by epigenetic alterations such as DNA methylation [145]. Anti-apoptotic genes *BCL2L2* and *E2F6* are targets of miR-205 and miR-31, respectively. By downregulating Bcl-w and E2F6, miR-205 and miR-31 promote chemotherapeutic agents-induced apoptosis in prostate cancer cells. The promoter region of the miR-205 gene was found to be hypermethylated in cell lines derived from advanced prostate cancers, contributing to the downregulation of the gene [146]. Moreover, miR-214 promoter is regulated by DNA methylation and reduces cell survival, induces apoptosis and enhances sensitivity to cisplatin through directly inhibiting Bcl2l2 expression in cervical cancer cells [147]. In many tumor types the promoters of the miR-34a and the miR-34b/c genes are subject to inactivation by CpG methylation. MiR-34a is commonly deleted in neuroblastomas. Furthermore, the loss of miR-34 expression has been linked to resistance against apoptosis induced by p53 activating agents used in chemotherapy [148]. 

## 5. Therapeutical Strategies

As seen above, methylation of several genes are associated with disease progression, survival, tumoral grade or metastasis and detection of these aberrant methylation in apoptosis genes could be used as markers of disease and constitute potential markers of prognosis in patients. Detection of such aberrant DNA methylation could be made in serum patients as illustrated in gastric cancers or in urine for bladder cancer patients [15,47,55,63,70,77,84,128,129,130,131,132,133,134,135,136,137,138,139,140,141,142,143,144,145,146,147,148,149]. Systematic research of methylation markers of disease in serum or urine might constitute a new non invasive approach for efficient detection and prognosis of cancer in the future.

Chemical agents targeting methylation machinery demonstrated positive effects against apoptosis resistance in both *in vitro* and *in vivo* studies. These interesting results lead to their proposal for clinical studies in cancer treatments. Natural compounds like green tea polyphenols such as EGCG have the potential to affect multiple biological pathways, including gene expression, growth factor-mediated pathways, the mitogen-activated protein kinase-dependent pathway, and the ubiquitin/proteasome degradation pathway [150]. The effects of dietary polyphenols such as EGCG on DNMTs appear to have their direct inhibition by interaction with the catalytic site of the DNMT1 molecule, and may also influence methylation status indirectly through metabolic effects associated with energy metabolism [151]. These bioactive components are able to modulate epigenetic events, and their epigenetic targets are known to be associated with breast cancer prevention and therapy [152,153]. Therapeutic activity of hypomethylating agents such as decitabine (DAC, Dacogen™; MGI Pharma, Inc.) and 5-azacytidine (AZA, Vidaza™; Celgene Corp.) have been used in clinical approaches in patients with myelodisplasic syndromes (MDS). Phase III clinical trials gave 60% of response in the arm treated with 5-aza and among them 7% of patients obtained a complete remission. Survival was increased from 14 months to 20 months. Similar studies were realized with decitabine, which gave a 17% response in a large phase III study. Global DNA hypomethylation induced by 5-azacytidine is progressive and related to cell cycle status [154]. Cells with large hypomethylation underwent apoptosis while cells without DNA hypomethylation were still alive. Several *in vitro* studies in different cancer cell lines demonstrated an efficient demethylation-dependent increase of pro-apoptotic proteins like DAPK and Bcl2L10 [22]. *In vitro* and *in vivo* studies in rodents demonstrated positive effects on combined decitabine and TRAIL death inducer on apoptosis sensibility of glioma cell lines mediated by both TRAILR1 and caspase-8 reexpression in a demethylation manner [155]. Moreover, a large portion of tumor-infiltrating CD8(+) T cells are FasL(+), and a critical role for FasL in decitabine and vorinostat-mediated tumor suppression *in vivo* is suggested. These data imply that combined modalities of chemotherapy to sensitize the tumor cell to Fas-mediated apoptosis and CTL immunotherapy is an effective approach for the suppression of colon cancer metastasis [156]. On the other hand, global DNA methylation and local hypomethylation in oncogenes or genes favoring apoptosis resistance argue in the addition of lipotropes (methionine, choline, folate, VitB12) as methyl donors to both prevent and limit aggressiveness. Treatment of glioma cells with folate reduced global hypomethylation, proliferation index and increased apoptosis sensitivity [157]. Similar results were observed in breast cancer cell lines [158]. Moreover, a delay in occurrence and a decrease in tumor size was observed in glioma chemo-induced rats treated with folate compared to normal diet [159]. Several mechanisms could explain these results. Invalidation of Dnmt1 has been associated to global DNA methylation, chromosal instability and lymphomas [160]. As a partial cooperation between *de novo* and maintaining Dnmts have also been reported increasing methyl donors accessibility may palliate mMase defects in cancers mainly promoted by Dnmt1/PCNA/URHF1 disruption and limit further chromosomal instability and local hypomethylations. Increasing dnMase activity is also supported by an increase in both Dnmt3a and 3b genes expression in glioma cells treated with folate in response to detachment of SP1 in favor of SP3 in these promoters. 

## 6. Conclusions

**Figure 3:**
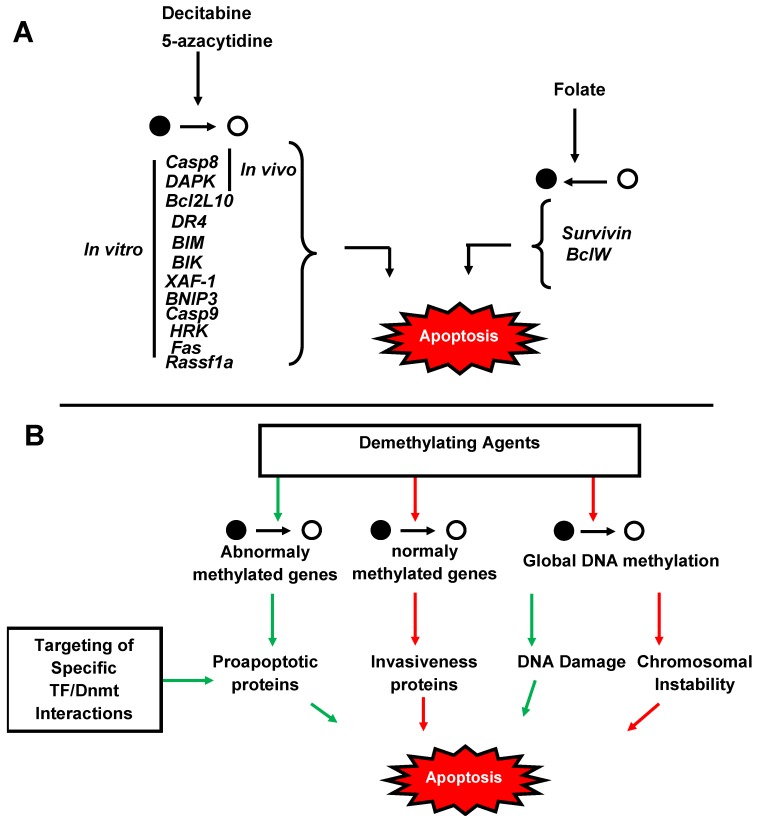
DNA methylation and anti-apoptotic strategies (**a**) Effects of both DNA demethylating agents and pro-methylation, mediated by folate, on apoptosis-related genes and apoptosis. (**b**) Conflicting results of demethylated agents and specific strategies against targeted DNA methylation on apoptosis.

Recent findings on targeted DNA methylation open new perspectives for therapeutical studies. Implication of c-Myc, p53 and SP1 in aberrant methylation of gene coding for pro-apoptotic proteins has to be more investigated. Systematic analysis of potential TF/Dnmt interaction revealed by *in vitro* studies may also reveal new specific interactions implicated in apoptosis resistance in cancer cells. By blocking these interactions, we could imagine both restore a specific demethylation of selective genes in cancer cells and apoptosis sensibility. This strategy would conturn significant side-effects currently produced by unspecific drugs like demethyl agents. While 5-aza treatment reduced apoptosis resistance in several cancer cell lines, prolonged exposition is associated with global DNA hypomethylation and activation of invasiveness pathway genes like UVA both *in vitro* and *in vivo* [161]. Combinations of existing therapies towards proliferative cells and specific reactivation of apoptosis sensitivity would constitue new therapies being more adapted to each patient in the future (Figure 3).
